# Adaptive evolution resulted in three subtypes of the *Riemerella anatipestifer crpR1* gene

**DOI:** 10.1128/jb.00156-26

**Published:** 2026-05-14

**Authors:** Jialing Wang, Xiaoli Du, Hui Yin, Xiaoying Zhang, Shiqi Wang, Hongyan Liao, Xingyu Zhang, Yuting Zheng, Qinghai Hu

**Affiliations:** 1 Shanghai Veterinary Research Institute, Chinese Academy of Agricultural Sciences 118161 , Shanghai, China

**Keywords:** *Riemerella anatipestifer*, *crpR1*, virulence, evolution, mutation

## Abstract

The global transcription regulator CRP protein controls diverse cellular processes in many bacteria. Our group previously reported that Tn4531 insertion in FIP52_09345 (designated as *crpR1*), which encodes a special CRP protein without the HTH motif, attenuated the virulence of *Riemerella anatipestifer*. Here, sequence analyses indicate that *R. anatipestifer crpR1* can be classified into three subtypes (*crpR1a*, *crpR1b*, and *crpR1c*) based on the ORF length. Of 52 strains, 42 carried *crpR1a*. CrpR1b and CrpR1c contained both the CRP domain and HTH motif, while CrpR1a lacked the HTH motif. The *crpR1a* sequence had two alleles, and *crpR1b* of some strains originated from allele 1 of *crpR1a*, while *crpR1c* of strain CH3 originated from allele 2 of *crpR1a*. Point mutations and insertion sequences may play an important role in the evolution of *crpR1*. Moreover, deletion of *crpR1a* from strain Yb2 resulted in reduced virulence in ducklings by approximately 1,000-fold, while deletion of the 3′-end of *crpR1a* (encoding the disordered region) or the downstream AS87_RS10485 (encoding the HTH motif) resulted in reduced virulence of strain Yb2 in ducklings. In addition, mutation of 450C451A in the *crpR1a* ORF of strain Yb2 to 450G generated the non-pathogenic Yb2 crpR1-450G mutant, with the *crpR1* subtype changed to *crpR1b*. Mutants with the 3′ end of the *crpR1* ORF in Yb2 crpR1-450G, consistent with that of strain HXb2 or CH3 *crpR1,* were pathogenic to ducklings. These findings confirm that adaptive evolution resulted in three subtypes of *R. anatipestifer crpR1*.

**IMPORTANCE:**

Bacterial CRP regulators regulate metabolism, stress resistance, biofilm formation, and pathogenesis. Here, we report that a special CRP in *Riemerella anatipestifer*, CrpR1a, which lacks the HTH motif, has biological functions. The *crpR1a,* carried by most *R. anatipestifer* strains, is the ancestor of *crpR1b* and *crpR1c*. Deletion of *crpR1a* in strain Yb2 or mutation of 450C451A to 450G in this *crpR1a* ORF leads to significant reduction or loss of virulence, but a single base mutation of 559C > A, 586C > T, or 591T > C in the non-pathogenic mutant Yb2 crpR1-450G *crpR1* ORF increases its virulence in ducklings. Moreover, deletion of the C-terminal disordered region of CrpR1a or its downstream HTH motif resulted in reduced virulence of strain Yb2.

## INTRODUCTION

*Riemerella anatipestifer* is the type species of the genus *Riemerella* in the newly recognized family *Weeksellaceae* within the order *Flavobacteriales* of the phylum *Bacteroidetes* (1). *R. anatipestifer*, which primarily infects domestic ducks, geese, and chickens, has been linked to significant economic losses in the duck industry worldwide. Upon infecting a duck flock, *R. anatipestifer* can become endemic and difficult to eradicate, potentially leading to repeated infectious episodes (2). To date, 21 *R. anatipestifer* serotypes have been identified, with notable variations in virulence, even within a given serotype (3). However, little is known about the mechanism underlying the pathogenesis of *R. anatipestifer*.

Our group previously identified 17 genes, including FIP52_09345 (AS87_RS10695 in strain Yb2, designated as *crpR1*), as essential for the survival and pathogenesis of *R. anatipestifer* in ducklings by signature-tagged transposon mutagenesis based on transposon Tn4351 (4). The *crpR1* gene of strain Yb2 encodes a putative Crp/Fnr family transcriptional regulator, which carries the conserved cyclic adenosine monophosphate receptor protein (CRP) domain (COG0664). The CRP protein is a global regulator in bacteria that plays a critical role in environmental adaptation, metabolism of sugars and amino acids, stress resistance, transport processes, biofilm formation, toxin production, and pilus synthesis (5–8). The regulatory roles of CRPs in virulence have been extensively studied in *Escherichia coli* (9–12), *Salmonella* (13, 14), *Yersinia pestis* (15), *Klebsiella pneumoniae* (16), *Vibrio cholerae* (17), and *Pseudomonas aeruginosa* (18).

Structurally, classical Crp/Fnr protein is composed of a C-terminal helix-turn-helix (HTH) motif that fits the DNA major groove and an N-terminal nucleotide-binding CRP domain (19). The CRP domain of *E. coli* is mostly responsible for subunit-subunit dimerization and binding of cyclic adenosine monophosphate, while the HTH motif is responsible for recognition and binding to DNA via regulators at the promoter region of target genes, thus functioning as an activator or repressor (6, 20). The transcriptional activation function of CRP involves binding to DNA (21), although the HTH motif is sometimes weakly conserved (6).

Four *R. anatipestifer* strains (Yb2, WJ4, CH3, and HXb2) were isolated from diseased ducklings in Jiangsu or Anhui Province, China, in 2000, but the lengths of the *crpR1* open reading frames (ORFs) of these strains differed (540, 540, 564, and 594 bp, respectively), suggesting evolutionary diversity. Unlike classical CRP proteins of other bacteria, strains Yb2 and WJ4 carried the CRP domain of CrpR1, but not the HTH motif, while CrpR1 of strains CH3 and HXb2 carried both. Therefore, *crpR1* was annotated as a pseudogene in early genome annotations of some *R. anatipestifer* strains in the GenBank database (https://www.ncbi.nlm.nih.gov/genbank/).

Therefore, the aims of this study were to determine why most *R. anatipestifer* strains do not contain the HTH motif and whether the CrpR1 protein without the HTH motif can function in regulating the virulence of *R. anatipestifer* by analyzing the evolution of the *crpR1* gene of *R. anatipestifer*.

## RESULTS

### *The R. anatipestifer crpR1* gene is classified into three subtypes based on deletion of one or two bases from the ORF

**Fig 1:**
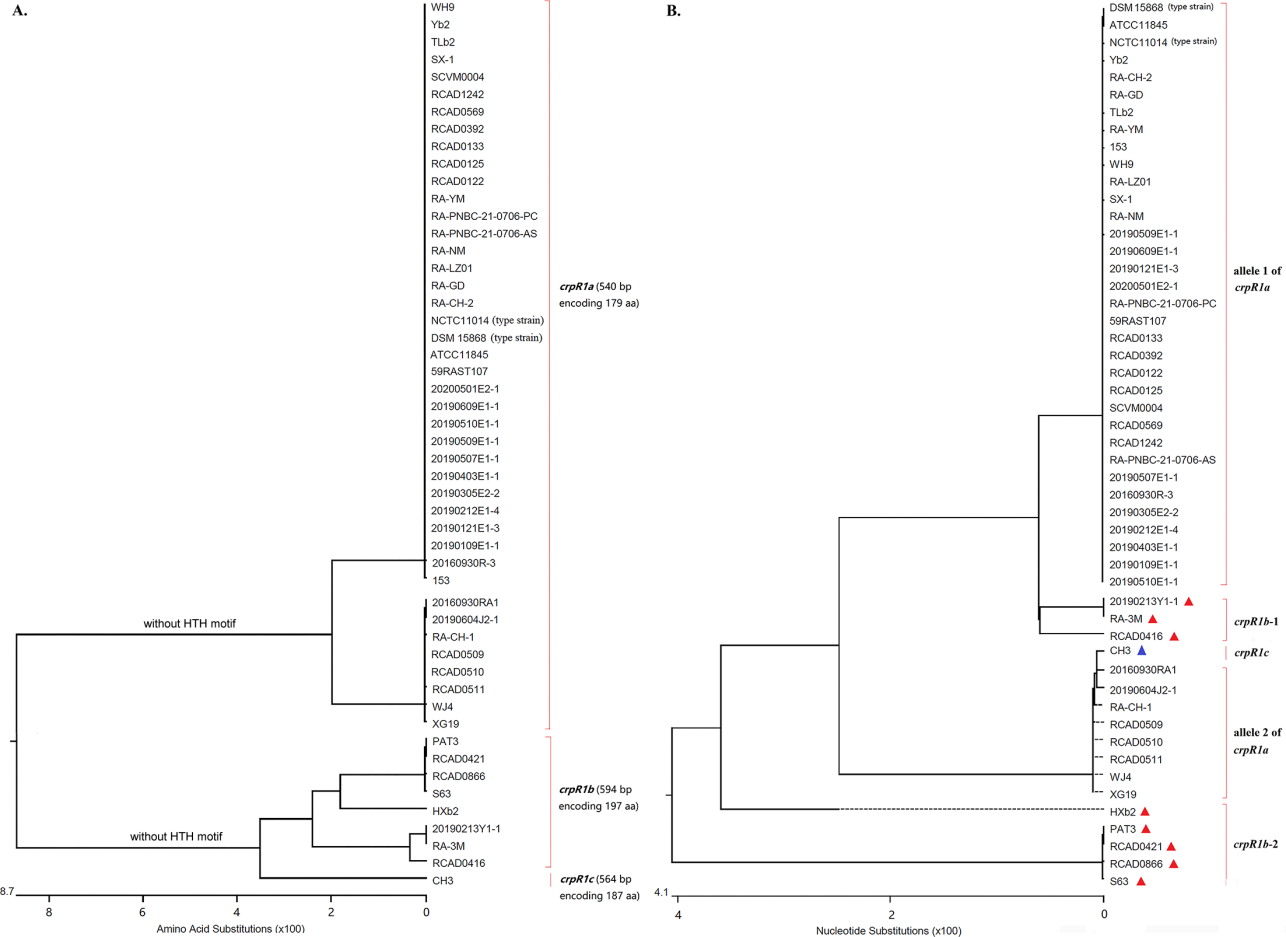
Phylogenetic tree of the *crpR1* ORF of *R. anatipestifer* strains. (**A**) The *crpR1* gene of *R. anatipestifer* can be classified into three subtypes (*crpR1a*, *crpR1b*, and *crpR1c*) based on the ORF length. (**B**) Phylogenetic tree of the *crpR1* ORF of 51 *R. anatipestifer* strains. Homology of the *crpR1* ORF of 51 *R. anatipestifer* strains as determined by alignment using the Clustal W method of the MegAlign module of the Lasergene v.7.1 software package (DNASTAR). Phylogenetic trees were generated from the resulting alignment.

Although the CrpR1 protein of *R. anatipestifer* strains Yb2 and WJ4 does not have an HTH motif, there is a small (132 bp) downstream ORF (AS87_RS10485) encoding the HTH motif that overlaps the 3′ end of the *crpR1* gene by 77 bases (Fig. S1). The homologous DNA fragments of the *crpR1* ORF of strain Yb2 plus the downstream AS87_RS10485 (595 bp) sequence were compared with the Basic Local Alignment Search Tool (BLAST; https://blast.ncbi.nlm.nih.gov/Blast.cgi). The *crpR1* gene sequences of 51 *R. anatipestifer* strains were retrieved from the GenBank database and aligned with the MegAlign program of the Lasergene 7.01 software package (DNASTAR, Inc.). The genome of all *R. anatipestifer* strains carried the *crpR1* gene. Of 51 *R. anatipestifer* strains, 42 (82.35%), including strains Yb2 and RA-CH-2, and the type strain ATCC 11845, had a *crpR1* ORF length of 540 bp, encoding 179 amino acids. Of these, 8 (19.05%) strains, including HXb2, S63, and RCAD0416, had a *crpR1* ORF of 594 bp, encoding 197 amino acids, while strain CH3 had a *crpR1* ORF length of 564 bp, encoding 187 amino acids. Therefore, as shown in Fig. 1A, the *R. anatipestifer crpR1* gene can be divided into three subtypes based on the ORF length: *crpR1a* (540 bp), *crpR1b* (594 bp), and *crpR1c* (564 bp). Similar to strains WJ4 and Yb2, the strains carrying the *crpR1a* gene had a small downstream ORF that encoded the HTH motif, which overlapped with the 3′ end of *crpR1a* ORF by 77 bases. Furthermore, the *crpR1a* ORF had two alleles, differentiated by 23 base substitutions (seven amino acids) primarily located at the 5′ end (Fig. S2), and among 42 *crpR1a*-carrying *R. anatipestifer* strains, 34 strains (81.0%) had 100% nucleotide homology in the crpR1a ORF, belonging to allele 1, and the other eight strains belonged to allele 2 (Fig. 1B). Moreover, based on nucleotide homology, *crpR1b* can also be further classified into *crpR1b*-1 and *crpR1b*-2 (Fig. 1B).

**Fig 2:**
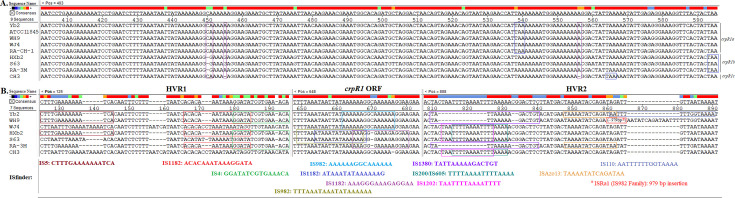
Comparative analysis of three subtypes of *crpR1* ORFs and their upstream and downstream sequences. (**A**) Deletion of one or two bases from the *crpR1* ORF in *R. anatipestifer* strains resulted in three *crpR1a* subtypes. Compared to *crpR1a* of strains Yb2, ATCC 11845, and others, 540C and 541A of the *crpR1* ORF were replaced with 540G in *crpR1b* of strain HXb2 and others, and 541A and 556A were deleted from *crpR1c* of strain CH3. (**B**) Two hypervariable regions (HVR1 and HVR2) were found upstream and downstream of the *crpR1* gene ORF. As compared to Yb2 and type strain ATCC 11845, multiple base insertions and substitutions were identified, and the HVR1 and HVR2 regions contained some insertion sequences (ISs), including a full ISRa1 insertion in the HVR2 of strain WH9.

As compared to *crpR1a* of strains Yb2 and ATCC 11845, the 540C and 541A nucleotides of the *crpR1b* ORF were replaced with 540G (e.g., HXb2 and other strains), while 455A and 556A were deleted from *crpR1c* of strain CH3 (Fig. 2A). One or two nucleotide deletions led to a frameshift in the *crpR1a* ORF and increased the length of the ORF, thus generating *crpR1*b and *crpR1c*, respectively (Fig. 2A). The CrpR1b and CrpR1c proteins of *R. anatipestifer* strains contained both the CRP domain and HTH motif of the classical CRP protein, while the CrpR1a protein only had the CRP domain (Fig. S3A).

### Sequence analysis revealed evolution of *R. anatipestifer crpR1* and neighboring genes

The type strain ATCC 11845 (DSM 15868; NCTC 11014), isolated in the United States from a duck in 1954 (22), is the earliest surviving strain of *R. anatipestifer*. Of 51 *R. anatipestifer* strains with full genomes in the GenBank database (up to 1 January 2025), 46 (90.2%) were isolated in China, and almost all were isolated within the past 30 years (Table S1). The *crpR1* of the type strain ATCC 11845 belongs to *crpR1a* (allele 1), suggesting that *crpR1a* may be the earliest *crpR1* subtype. A phylogenetic tree of the *R. anatipestifer crpR1* ORF showed that *crpR1* of strains RA-3M, RACD0416, and 20190213Y1-1, which belong to *crpR1b*-1, are closely related to allele 1 of *crpR1a*, and that CH3 (*crpR1c*) is more closely related to allele 2 of *crpR1a* (Fig. 1B), indicating that *crpR1* of strains RA-3M, RACD0416, and 20190213Y1-1 (*crpR1b*-1) originated from allele 1 of *crpR1a*, and that CH3 originated from allele 2 of *crpR1a*. Allele 1 was present in 81% (34/42) of *crpR1a*-carrying *R. anatipestifer* strains, including the earliest strain ATCC 11845, suggesting that allele 2 of *crpR1a* may have originated from allele 1.

To investigate how *crpR1* of *R. anatipestifer* evolved from the earliest *crpR1a* (e.g., type strain ATCC 11845) to *crpR1b* and *crpR1c*, the neighboring genes of *crpR1* and the lengths of the intergenic regions of strains Yb2, HXb2, and CH3 were compared. A BLAST search of a 6,120 bp DNA sequence of strain Yb2 showed significant differences in the lengths of the ORFs and intergenic regions of neighboring genes in other *R. anatipestifer* strains, and many base substitutions, deletions, or insertions in the regions carrying *crpR1b* or *crpR1c* (data not shown), especially within 200 bp upstream and downstream of *crpR1*. Then, 951 bp of *crpR1* and the neighboring nucleotide sequences of all 51 *R. anatipestifer* strains retrieved from the GenBank database were aligned using the MegAlign program (Lasergene). As compared to that of strain Yb2 and the type strain ATCC 11845 (*crpR1a*), there were two hypervariable regions (HVRs), HVR1 (nucleotides 128–180) and HVR2 (nucleotides 795–840), corresponding to 951 bp of Yb2 *crpR1* and the neighboring sequences located upstream and downstream of the *crpR1* gene ORF (nucleotides 206–745), respectively (Fig. 2B). Multiple base insertions and substitutions appeared in HVR1 and HVR2, indicating that these two regions were hotspots of DNA mutations.

To determine whether insertion sequences (ISs) or DNA repeats play a role in base mutations in these two hotspot regions and within the *crpR1* ORF, 951 bp (*crpR1* ORF and the neighboring sequence) of strains Yb2 and WH9 (carrying *crpR1a*), HXb2 (carrying *crpR1b*), and CH3 (carrying *crpR1c*) were further searched against the ISfinder database and the repeats-finder tool to identify ISs and repeat sequences. The results identified some short ISs, including IS1182, IS982, IS4, and IS5, in the HVR1 and HVR2 regions of *crpR1* (Fig. 2B). Notably, the 979 bp ISRa1 (IS982 family) contained an ORF of 879 bp encoding a transposase inserted into the HVR2 region of strain WH9, and ISRa1 was widely distributed among *R. anatipestifer* strains, as demonstrated by the BLAST results (data not shown). Moreover, two short DNA ISs (IS982 and IS1182) were also found at the 450G mutation site of the *crpR1* ORF of strains Yb2 and HXb2, suggesting that the 450G site of the *crpR1* ORF of strain HXb2 (and other *crpR1b* sequences) may also be mutated from 450C451A of the *crpR1a* ORF through IS insertion or recombination, although other factors cannot be ruled out. In addition, some short repeats were also found in these regions, and whether these short repeats were involved in mutations in the *R. anatipestifer crpR1* ORF and the flanking sequences remains to be clarified. Collectively, these findings indicate that ISs play an important role in the evolution of *CrpR1* and neighboring genes.

### CrpR1 is involved in the virulence of *R. anatipestifer* strain Yb2

**Fig 3:**
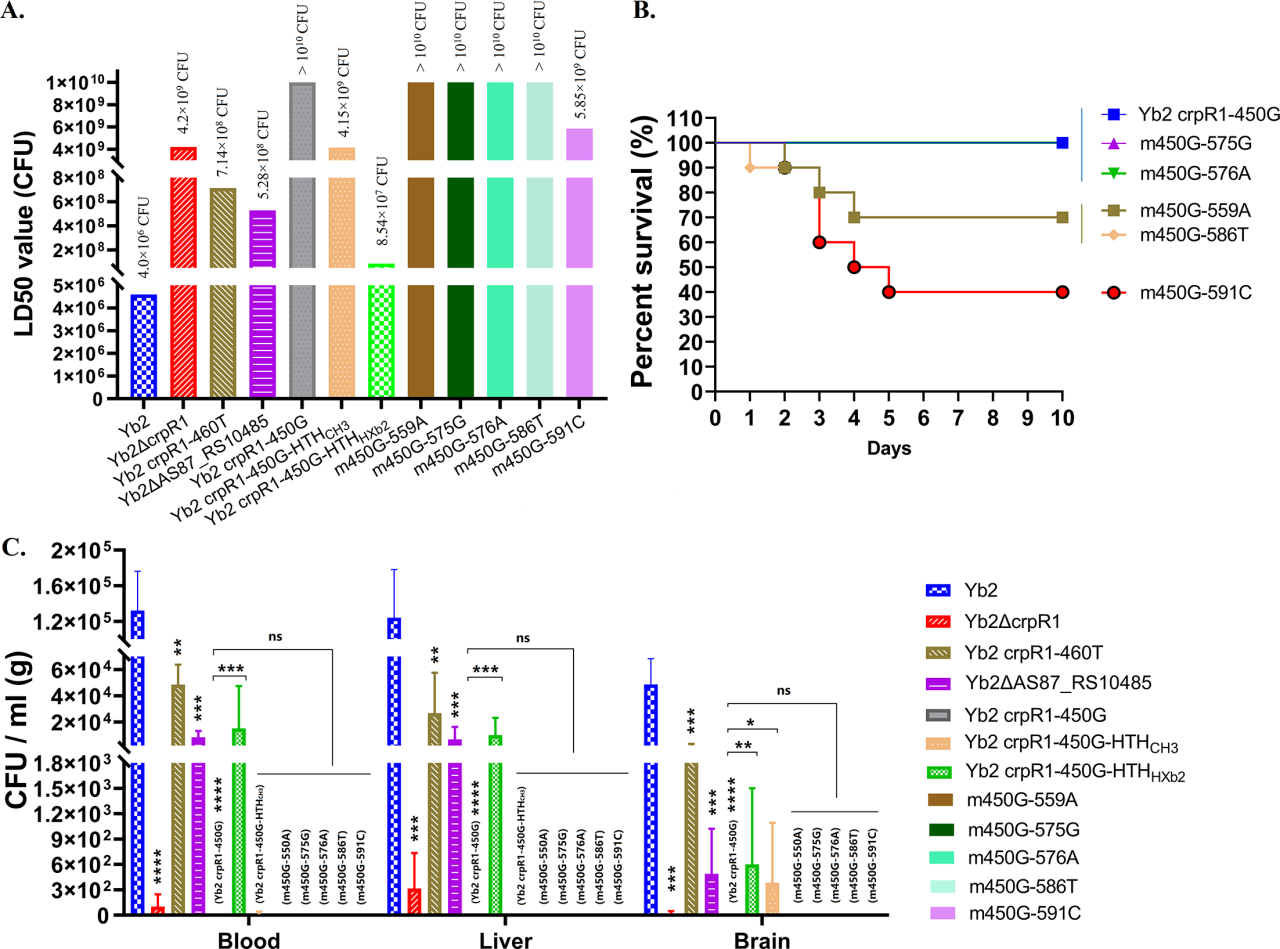
The pathogenicity of the wild-type Yb2, the deletion mutant Yb2ΔcrpR1, and mutant strains with point mutations in ducklings. (**A**) The median lethal dose (LD_50_) of the wild-type Yb2, the deletion mutant Yb2ΔcrpR1, and 10 mutants with point mutations in ducklings. (**B**) Survival rate of Yb2crpR1-450G and its derived mutants with five single base mutations in ducks inoculated with a dose of 10^10^ CFU. (**C**) Bacterial loads in blood, liver, and brain tissues of ducklings infected with the wild-type Yb2, the deletion mutant Yb2ΔcrpR1, and 10 mutant strains with point mutations. For each strain, eight ducklings were intramuscularly inoculated with 1 × 10^8^ CFU. At 24 h post-inoculation, blood, liver, and brain samples were collected from the inoculated ducklings, and the bacterial loads were determined by plate counts. Data are presented as the mean ± standard deviation of eight infected ducklings. Asterisks indicate statistically significant differences between two groups (*P < 0.05; **P < 0.01; ***P < 0.001; ****P < 0.0001; ns, no significant difference; asterisks that do not directly indicate the comparison group refer to the comparisons with the wild-type Yb2).

Our group previously reported that insertion of the transposon Tn4351 into the *crpR1* sequence of *R. anatipestifer* strain WJ4 resulted in reduced virulence in ducklings (4). To confirm the role of CrpR1 in the virulence of *R. anatipestifer*, *crpR1* of the virulent strain Yb2 was in-frame deleted by homologous recombination using a recombinant suicide plasmid to generate the deletion mutant Yb2ΔcrpR1. There was no significant difference in the growth curves between the mutant Yb2ΔcrpR1 and the parent strain Yb2 (Fig. S4A). Duck infection experiments showed that the LD_50_ of the mutant Yb2ΔcrpR1 in 8-day-old ducklings was 4.2 × 10^9^ colony-forming units (CFU), which was about 10^3^-fold greater than that of the parent strain Yb2 (4.0 × 10^6^ CFU, Fig. 3A). In addition, the bacterial loads in blood, liver, and brain tissues of ducklings infected with the mutant Yb2ΔcrpR1 were significantly decreased as compared to those of ducklings infected with the parent strain Yb2 at 24 h post-inoculation (Fig. 3B). These findings demonstrate that CrpR1 is involved in the virulence of strain Yb2.

### Mutation of 450C451A to 450G in the *crpR1* ORF of strain Yb2 leads to loss of virulence

Replacing 540C and 541A of the *crpR1* ORF (*crpR1a*) sequence of strain Yb2 with a single G nucleotide resulted in a mutant (designated as Yb2 crpR1-450G) of 594 bp, with the encoded protein containing both the CRP domain and HTH motif (Fig. 2A and Fig. S3A), sharing 97.0% nucleotide and amino acid homology with *crpR1b* of strain HXb2. To investigate why most (42/51, 82.35%) of the investigated *R. anatipestifer* strains carry *crpR1a*, but not *crpR1b* or *crpR1c*, the mutant Yb2 crpR1-450G was constructed by mutating 450C451A to 450G (Fig. S5A) using a recombinant suicide plasmid, which resulted in a change of the *crpR1* subtype from *crpR1a* (540 bp) to *crpR1b* (594 bp). The growth of Yb2 crpR1-450G showed no significant difference compared to the wild-type Yb2 (Fig. S4A). Subsequent analysis showed that the mutant Yb2 crpR1-450G was not pathogenic to 8-day-old Cherry Valley ducklings with an LD_50_ ≥10^10^ CFU, which was more than 2,500-fold greater than the pathogenicity of the parent strain Yb2 (4.0 × 10^6^ CFU, Fig. 3A). Moreover, the blood, liver, and brain tissues of ducklings infected with the mutant Yb2 crpR1-450G were free of *R. anatipestifer* (Fig. 3C). These results also suggest that CrpR1 plays an important role in the pathogenesis of *R. anatipestifer*. The loss of pathogenicity of the mutant Yb2 crpR1-450G could explain why the mutant strain was not isolated from infected ducklings and why the *crpR1* ORF of most of *R. anatipestifer* strains exists in the form of the 540 bp *crpR1a* mutant rather than Yb2 crpR1-450G-like *crpR1b* gene.

### The 3′ end mutation of the *crpR1* ORF enhances the pathogenicity of Yb2 crpR1-450G

To investigate why *R. anatipestifer* strains HXb2 and CH3, which share a highly homologous C-terminal of the CrpR1 protein with the mutant Yb2 crpR1-450G (Fig. S5A), are pathogenic to ducklings (23, 24), but not the mutant Yb2 crpR1-450G, the 3′ end of the *crpR1* ORF of strain Yb2 crpR1-450G was mutated by homologous recombination using a recombinant suicide plasmid to be identical to that of *crpR1* of strain HXb2 or CH3 (Fig. S5B), to generate the mutants Yb2 crpR1-450G-HTH_HXb2_ and Yb2 crpR1-450G-HTH_CH3_, in which 555A was deleted at the *crpR1* ORF of Yb2 crpR1-450G (Fig. S5B). The results of duck challenge experiments showed that the LD_50_ of Yb2 crpR1-450G-HTH_HXb2_ and Yb2 crpR1-450G-HTH_CH3_ was 8.54 × 10^7^ and 4.15 × 10^9^ CFU, respectively (Fig. 3A), and the bacterial loads in blood, liver, and brain tissues of ducklings infected with Yb2 crpR1-450G-HTH_HXb2_ were significantly higher than those of ducklings infected with Yb2 crpR1-450G, but there were no significant differences in the bacterial loads of ducklings infected with 10^8^ CFU of Yb2 crpR1-450G-HTH_CH3_ and Yb2 crpR1-450G (Fig. 3C). These results indicate that strain Yb2 crpR1-450G can be transformed from non-pathogenic to pathogenic by point mutations or one-base deletion at the 3′ end of the *crpR1*-450G ORF. Moreover, the LD_50_ of Yb2 crpR1-450G-HTH_HXb2_ in ducklings was about 48-fold lower than that of Yb2 crpR1-450G-HTH_CH3_. Nucleotide sequence analysis showed that five point mutations at the 3′ end of *crpR1* generated in the mutant Yb2 crpR1-450G-HTH_HXb2_ were the common nucleotide replacements between *crpR1b* and *crpR1a* (Fig. S6). To further explore which of the five mutated bases plays a critical role in the transformation of the non-pathogenic strain Yb2 crpR1-450G into a pathogenic strain, five single base mutants m450G-559A, m450G-575G, m450G-576A, m450G-586T, and m450G-591C were constructed using Yb2 crpR1-450G as the parent strain (Fig. S5C). The single base mutation at these sites did not affect the growth of strain Yb2 crpR1-450G (Fig. S4B). Animal infection experiments showed that the LD50s of these five mutants were above 10^10^ CFU (Fig. 3A), and no bacterial loads were detected in ducklings infected with 10^8^ CFU of each mutant (Fig. 3C). However, 60% (6/10), 30% (3/10) and 30% (3/10) ducklings challenged with 10^10^ CFU of m450G-591C, m450G-586T, and m450G-559A, respectively, were dead, while no ducklings infected with 10^10^ CFU of Yb2 crpR1-450G were dead (Fig. 3B), indicating that the 591T > C, 586C > T, and 559C > A mutations play important roles in the evolution of non-pathogenic Yb2 crpR1-450G, or Yb2 crpR1-450G-like strains into pathogenic strains.

### Deletion of the disordered region of *crpR1* or mutations of the start codon of the downstream AS87_RS10485 reduced virulence of strain Yb2 in ducklings

As described above, CrpR1a from 42 (82.35%) of 51 *R. anatipestifer* strains did not have an HTH motif, but rather the small ORF AS87_RS10485 encoding the HTH motif. Moreover, a BLAST search of the conserved domains and three-dimensional (3D) structures of CrpR1 of strains Yb2 and HXb2 against the Universal Protein Resource (UniProt) website showed that the C-terminal of the CrpR1a protein, which is involved in the virulence of strain Yb2, contained a disordered region (amino acid residues 154–179) and there was a significant difference in the 3D structures between CrpR1a of strain Yb2 and CrpR1b of strain HXb2 (Fig. S3B).

To explore the role of the C-terminal of CrpR1a in virulence regulation of strain Yb2, the C-terminal disordered region of CrpR1 of strain Yb2 was deleted by introducing two stop codons into the *crpR1* ORF by mutating 460A to 460T and inserting a T nucleotide between positions 462 and 463, which generated the mutant Yb2 crpR1-460T (Fig. S5D). Animal experiments showed that the LD_50_ of Yb2 crpR1-460T in ducklings was 7.14 × 10^8^ CFU, which is about 180-fold greater than that of wild-type Yb2 (Fig. 3A). Bacterial loads in the blood, liver, and brain tissues were significantly lower in ducklings infected with Yb2 crpR1-460T as compared to those of Yb2 (Fig. 3C). Moreover, an AS87_RS10485 deletion mutant of strain Yb2 was constructed to investigate the role of AS87_RS10485 in the regulatory function of the CrpR1a protein. There were three initiation codons (ATG) in the ORF of AS87_RS10485, which can form three ORFs of 132, 105, and 96 bp, with the same 3′ end. To avoid influencing expression of the gene downstream of AS87_RS10485 and the amino acids encoded by the overlapping portion of *crpR1*, the mutant Yb2ΔAS87_RS10485 was constructed by mutating the three ATG codons to ACG (Fig. S5E). The results of real-time PCR showed that the relative mRNA levels of AS87_RS10485 in the mutant Yb2ΔAS87_RS10485 were significantly reduced as compared to the parent strain Yb2 (*P* < 0.0001, Fig. S7). The LD_50_ of ducklings infected with Yb2ΔAS87_RS10485 was 5.28 × 10^8^ CFU (Fig. 3A), which was about 130-fold greater than that of wild-type Yb2, and the bacterial loads in blood, liver, and brain tissues were significantly lower (Fig. 3C). These results indicate that both the C-terminal disordered region of CrpR1a and HTH motif encoded by AS87_RS10485 are involved in regulating the virulence of *R. anatipestifer* strain Yb2.

## DISCUSSION

Bacteria can adapt to environmental changes through mutation followed by natural selection, a phenomenon known as adaptive evolution (25). For *R. anatipestifer*, only the mutants with the capacity to infect and proliferate in the hosts, such as ducks and geese, can survive. The results of the present study showed that the CRP protein without the HTH motif regulated the virulence of *R. anatipestifer,* and *crpR1* has three subtypes due to deletion of one or two bases in the ORF, and hotspot regions for mutations were found in the flanking sequences of the *crpR1* ORF, thereby revealing the evolution of *R. anatipestifer crpR1*.

Generally, point mutations, horizontal gene transfer (HGT), and genetic rearrangements are the major driving forces of bacterial evolution (26). Point mutations based on random mutagenesis are considered to drive the evolution of bacteria, leading to the modification, inactivation, or differential regulation of existing genes, which have certainly contributed to the diversification of bacteria on an evolutionary timescale (27). Many point mutations are patho-adaptive (28), which enable single bacterial clones to become pathogenic. Such patho-adaptive point mutations can explain how the non-pathogenic Yb2 crpR1-450G mutant evolved to the pathogenic mutants Yb2 crpR1-450G-HTH_HXb2_ and Yb2 crpR1-450G-HTH_CH3_ through base substitutions or one-base deletion at the 3′ end of Yb2 crpR1-450G, which allowed isolation of *R. anatipestifer* strains HXb2 and CH3 from naturally infected dead ducks, but not Yb2 crpR1-450G-like strains. Moreover, as far as we know, HXb2, which carries the *crpR1b* gene, is currently the most pathogenic *R. anatipestifer* strain in ducklings (24). The LD_50_ of Yb2 crpR1-450G-HTH_HXb2_ was about 48-fold lower than that of Yb2 crpR1-450G-HTH_CH3_, although the link to the greater pathogenicity of strain HXb2 as compared to strain CH3 remains unclear. In addition, our results indicate that the *R. anatipestifer crpR1* gene can be classified into three subtypes based on the deletion of one or two bases within the ORF, demonstrating that point mutations may be involved in these base deletions. Point mutations can also occur at other bases of the *crpR1* ORF, especially at the 5′ end, resulting in a switch from *crpR1a* allele 1 to allele 2, and at the nucleotide sites upstream and downstream of the *crpR1* ORF. However, it is difficult to account for the ability of bacteria to exploit new environments by the accumulation of point mutations alone (27).

HGT plays an integral role in the evolution, diversification, and speciation of bacteria (27), and is central to bacterial adaptation, which is facilitated by mobile genetic elements (MGEs), such as ISs, tandem or inverted repeats, transposable elements, bacteriophages, plasmids, integrons, and genomic islands (29, 30). The three classic mechanisms for HGT are transformation by naked DNA, transduction mediated by bacteriophages, and conjugation allowing the transfer of DNA fragments, transposons, or plasmids among cells (31, 32). Homologous recombination mediated by RecA can result in substitution of only a few nucleotides in regions with high sequence similarity or often the integration of additional DNA sequences (33). Homologous recombination and HGT are tightly interconnected (34), whereas transposable elements, including typical ISs, miniature inverted-repeat transposable elements, transposons, composite transposons, conjugative transposons, and transposable bacteriophages, are DNA sequences with defined ends that can move within and between genomes by means of excision and integration reactions independent of homologous recombination (35). Thus, mutations to *crpR1* and flanking sequences in one *R. anatipestifer* strain may be from another *R. anatipestifer* strain or other *Bacteroides* through HGT with or without homologous recombination.

ISs can occur in a wide range of copy numbers in a particular bacterial genome and can move within the genome (between different DNA molecules or within an individual DNA molecule) or horizontally between genomes as part of other MGE vectors, such as phages and plasmids (36). DNA sequencing has been used to identify HGT (27). In this study, some ISs were located in the hotspot regions HVR1 and HVR2 upstream and downstream of the *crpR1* ORF. The full IS element IS*Ra1,* including a 15 bp imperfect repeat and an 879 bp ORF encoding transposase responsible for catalyzing DNA cleavages and strand transfer necessary for transposition (37), was inserted into the HVR2 region of *crpR1* of *R. anatipestifer* strain WH9 (GenBank accession number: CP033039.1). IS*Ra1* was initially identified on the plasmid pCFC2 in *R. anatipestifer* (37) and widely distributed throughout the genomes and plasmids of various *R. anatipestifer* strains, indicating that ISs are important drivers of the evolution of *R. anatipestifer crpR1*. Besides plasmids (37, 38)*, R. anatipestifer* bacteriophages were also isolated from the feces of healthy Muscovy ducks in China (39), which were integrated into the chromosome of *R. anatipestifer (40*), demonstrating that plasmids and phages may act as MGE vectors in *R. anatipestifer*. In addition, homologous recombination can also occur when multiple copies of an IS are carried by a chromosome or plasmid, with a variety of consequences, such as deletions, inversions, and duplications. Moreover, deletion sites of the bacterial chromosome are often flanked by either perfect or imperfect repeats, which may play a role in illegitimate recombination (26).

Repetitive regions of the genome often serve as hotspots of rearrangements and evolution, and repetitive loci are prone to recombination, producing insertion/deletions (indels) and translocations between both adjacent and distal repeats (35). In addition, ISs also often generate short flanking direct repeat sequences at the integration site using the cohesive end of the DNA, and some IS elements generate completely conserved or variable direct repeat sequences (41, 42). In this study, some short repeats were found in *crpR1* and related flanking regions, suggesting that repeats may also be involved in the evolution of *crpR1*. In addition, some repeats may have also been derived from homologous recombination or point mutations, which result in a related DNA sequence identical to those of short IS and repeat sequences.

Besides mobile genetic elements, natural transformation also involves the integration of naked DNA from the extracellular environment into the bacterial genome (43). Natural transformation often occurs within populations of bacterial cells that are members of the same or closely related species, which provides a mechanism of diversification by generating mosaic genes through homologous recombination between similar existing alleles (32). Natural transformation can occur in *R. anatipestifer* by uptake of foreign DNA (44).

In addition, most of the investigated strains of *R. anatipestifer* carrying *crpR1a* may have resulted from involvement of both the disordered region and the downstream ORF encoding the HTH motif in the virulence of strain Yb2, leading to the regulatory function of CrpR1a, and the pathogenicity of Yb2 was stronger than most of the other *R. anatipestifer* strains. On the other hand, *crpR1a* is the ancestor of crpR1b and crpR1c. Only mutant strains that are pathogenic and have the capacity to proliferate in the host can survive, while the non-pathogenic mutants, such as the Yb2 crpR1-450G-like mutant, cannot be isolated from ducks or other birds. In addition, *R. anatipestifer crpR1a* has two alleles with 23 base substitutions and seven amino acid differences at the 5′ ends. A possible explanation why *crpR1a* mutations (allele 2) from these eight bacterial strains are consistent at these 23 base sites is that these eight strains may have originated from the same ancestor. Some factors may be drivers of the 23 base mutations of *crpR1a* allele 2, such as point mutations and ISs. Some short IS fragments were found at positions 1–170 of Yb2 and WJ4 *crpR1*. Notably, all *R. anatipestifer* strains carrying *crpR1b* and *crpR1c* in the GenBank database were isolated in China, which may be attributed to the fact that about 70% of the global duck population is raised in China, making *R. anatipestifer* infection a common disease, thereby accelerating genetic evolution of *R. anatipestifer*. In future studies, we plan to investigate how CrpR1 regulates the virulence of *R. anatipestifer*.

Taken together, these findings suggest that point mutations and HGT (ISs, bacteriophages, and plasmids) with or without homologous recombination may be involved in the evolution of *crpR1* in *R. anatipestifer*, and adaptive evolution has resulted in three subtypes of *crpR1*.

## MATERIALS AND METHODS

### Bacterial strains and growth conditions

*R. anatipestifer* serotype 2 strain Yb2 (23) and related derivatives were grown in tryptic soy broth (Becton, Dickinson and Company, Sparks, MD, USA) at 37°C or on tryptic soy agar at 37°C under an atmosphere of 5% CO_2_/95% air. *E. coli* strains were cultured in Luria-Bertani broth or agar at 37°C. Antibiotics (ampicillin, 100 μg/mL; erythromycin, 0.5 μg/mL; kanamycin, 50 μg/mL) were used when needed. The bacterial strains used in this study are listed in Table 1.

**TABLE 1:** Bacterial strains and plasmid used in this study^a^

Strain or plasmid	Characteristic(s)	Reference or source
Strains		
Yb2	A wild-type strain of serotype 2 *Riemerella anatipestifer*	(4)
Yb2ΔcrpR1	The *crpR1* gene deletion mutant of *R. anatipestifer* Yb2	This study
Yb2 crpR1-450G	The mutant in which 450C451A in the *crpR1a* ORF of strain Yb2 was mutated to a single base G	This study
Yb2 crpR1-450G-HTH_HXb2_	The mutant in which 3′ end of the *crpR1* ORF in Yb2 crpR1-450G was mutated to be consistent with that of HXb2 *crpR1* ORF	This study
Yb2 crpR1-450G-HTH_CH3_	The mutant in which 3′ end of the *crpR1* ORF in Yb2 crpR1-450G was mutated to be consistent with that of CH3 *crpR1* ORF	This study
Yb2 crpR1-460T	The mutant in which two consecutive stop codons were introduced at nucleotides 460–463 with 460A > T and 462_463insT in the *crpR1* ORF of strain Yb2	This study
Yb2 crpR1-mHTH	The mutant in which three ATGs in AS87_RS10485 ORF in strain Yb2 was mutated to ACG	This study
m450G-559A	A single base mutant in which the nucleotide 559C of *crpR1* ORF in Yb2 crpR1-450G was mutated to A	This study
m450G-575G	A single base mutant in which the nucleotide 575A of *crpR1* ORF in Yb2 crpR1-450G was mutated to G	This study
m450G-576A	A single base mutant in which the nucleotide 576G of *crpR1* ORF in Yb2 crpR1-450G was mutated to A	This study
m450G-586T	A single base mutant in which the nucleotide 586C of *crpR1* ORF in Yb2 crpR1-450G was mutated to T	This study
m450G-591C	A single base mutant in which the nucleotide 591T of *crpR1* ORF in Yb2 crpR1-450G was mutated to C	This study
*E. coli* S17-1	Lpir hsdR pro thi; chromosomal integrated RP4-2 Tc::Mu Km::Tn7	Biomedal
Plasmid		
pYT354	Suicide plasmid, Em^r^, Ap^r^.	(44)

^a^ Antibiotic resistance phenotypes are as follows: ampicillin, Ap^r^; erythromycin, Em^r^.

### Phylogenetic analysis and search of ISs and repeats

To study the evolutionary relationship of the *crpR1* gene among different *R. anatipestifer* strains, the *crpR1* ORF and the flanking sequences of 51 *R. anatipestifer* strains isolated from different regions worldwide in different years were retrieved from the GenBank database. The GenBank accession number of each strain used in this study is listed in Table S1. Multiple sequence alignments were performed using the ClustalW method of the MegAlign module of Lasergene v.7.1 software (DNASTAR, Inc., Madison, WI, USA) and used to generate phylogenetic trees.

The ISfinder database (https://www-is.biotoul.fr/blast.php) and repeats-finder tool (https://www.novopro.cn/tools/repeats-finder.html) were used to search ISs and repeats in the *crpR1* gene and flanking sequences of strains Yb2 and WH9 (carrying *crpR1a*), HXb2 (carrying *crpR1b*), and CH3 (carrying *crpR1c*).

### Predicted domain and protein structure

The conserved domains (CDs) of CrpR1 of *R. anatipestifer* strains Yb2, HXb2, and CH3 were searched against the UniProt database (https://www.uniprot.org/) using the NCBI CD-search tool (https://www.ncbi.nlm.nih.gov/Structure/cdd/wrpsb.cgi).

Enabled by the groundbreaking AlphaFold2 artificial intelligence system, the predictions archived in the AlphaFold database have been integrated into the UniProt Knowledgebase and other databases (45). More than 85% of UniProt entries contain a predicted protein structure, provided by AlphaFold, an artificial intelligence system developed by DeepMind to predict protein structures from amino acid sequences (46). The predicted three-dimensional (3D) structures of the CrpR1 proteins of *R. anatipestifer* strains Yb2 and HXb2 from the UniProt Knowledgebase were aligned using the BLAST algorithm and the AlphaFold system with default parameters based on the amino acid sequences of the CrpR1 proteins.

### Generation of mutant strains

The primers used in this study are listed in Table S2. Deletion of the *crpR1* gene from the chromosome of *R. anatipestifer* strain Yb2 was performed by homologous recombination using the suicide plasmid pYT354 (44), as described previously (47). Briefly, the upstream and downstream homology arms were amplified by PCR and then ligated into plasmid pYT354 using the ClonExpress Ultra One Step Cloning Kit (Vazyme, Nanjing, China) to generate the recombinant suicide plasmid pYT354-crpR1-LR, which was introduced into strain Yb2 by conjugation using *E. coli* S17-1 (pYT354-crpR1) as the donor and *R. anatipestifer* Yb2 cells as the recipient. Finally, the deletion mutant Yb2ΔcrpR1 was identified by PCR using a specific primer pair and further confirmed by DNA sequencing.

In this study, mutants with one or more base substitutions, insertions, or deletions were generated using homologous recombination of recombinant suicide plasmids as described above. The sequence with one or more base-site substitutions, insertions, or deletions was used as part of the PCR primer to amplify the homologous arms to introduce the mutation. The mutants identified by PCR were further confirmed by DNA sequencing. To construct the Yb2 crpR1-450G mutant, the nucleotide sequence 450451CA of the *crpR1* ORF of strain Yb2 was mutated to a single nucleotide (450G) (Fig. S5A). To construct a mutated strain with the 3′ end of the *crpR1* ORF in the Yb2 crpR1-450G mutant strain the same as the 3′ end of *crpR1* of strain HXb2, five nucleotides in the ORF were mutated (559C > A, 575576AG > GA, 586C > T, and 591T > C) to generate the mutant Yb2 crpR1-450G-HTH_HXb2_ (Fig. S5B). In addition, to further investigate which base was related to the virulence of the mutant Yb2 crpR1-450G-HTH_HXb2_, five single base mutants m450G-559A, m450G-575G, m450G-576A, m450G-586T, and m450G-591C were constructed using Yb2 crpR1-450G as the parent strain (Fig. S5C). To construct a mutant with the 3′ end of the *crpR1* ORF of the Yb2 crpR1-450G mutant strain the same as the 3′ end of *crpR1* of strain CH3, a single base (555A) was deleted from the ORF to generate the mutant Yb2 crpR1-450G-HTH_CH3_ (Fig. S5B). To construct the C-terminal disordered region deletion mutant of CrpR1 of strain Yb2, two consecutive stop codons were introduced into the *crpR1* ORF (460A > T and 462_463insT) to generate the mutant Yb2 crpR1-460T (Fig. S5D). To construct an AS87_RS10485 deletion mutant in strain Yb2, the three ATG sequences in the ORF were mutated to ACG while maintaining the C-terminal amino acid sequences of CrpR1, to generate the mutant Yb2ΔAS87_RS10485 (Fig. S5E).

### Assessment of the virulence of the mutants in ducklings

One-day-old Cherry Valley ducklings (Lijia Duck Farm, Taizhou, Jiangsu Province, China) were seronegative for the serotype 2 *R. anatipestifer* antibody, as confirmed with an indirect enzyme-linked immunosorbent assay using extracted lipopolysaccharides of strain Yb2 as the coating antigen (48). The ducklings were housed in cages at a controlled temperature of 24°C–26°C with free access to food and water.

To determine the virulence of the constructed mutants and parent strain Yb2, the median lethal dose (LD_50_) in ducklings was calculated as described previously (23). Briefly, for each strain, 8-day-old Cherry Valley ducklings were randomly divided into six groups of 10 ducklings each and intramuscularly challenged with 10^5^, 10^6^, 10^7^, 10^8^, 10^9^, or 10^10^ CFU, respectively. Ducklings that became moribund were humanely sacrificed and counted as dead. Dead ducklings were subjected to *R. anatipestifer* identification. The number of deceased ducklings was recorded daily for 10 days post-challenge. The LD_50_ was calculated using the Reed-Muench method (49). In addition, the bacterial loads in duckling blood, liver, and brain samples of the wild-type strain Yb2 and each mutant were measured. Eight-day-old Cherry Valley ducklings were intramuscularly injected with 10^8^ CFU of each strain and samples were collected from six ducklings in each group at 24 h post-inoculation and plated on tryptic soy agar plates for bacteria counting.

### Statistical analysis

Prism 9.0.0 software (GraphPad Software, Inc., San Diego, CA, USA) was used for statistical analysis and preparation of graphs. Significant differences between groups were identified using the two-tailed Student’s *t*-test. A probability (*P*) value of <0.05 was considered statistically significant.

## SUPPLEMENTAL MATERIAL

Supplemental figures and tables jb.00156-26-s0001.pdf Fig. S1 to S7, and Tables S1 and S2.

ASM does not own the copyrights to Supplemental Material that may be linked to, or accessed through, an article. The authors have granted ASM a non-exclusive, world-wide license to publish the Supplemental Material files. Please contact the corresponding author directly for reuse.
