# Supplementary material for: Adaptive evolution resulted in three subtypes of the Riemerella anatipestifer crpR1 gene
Source: J Bacteriol. 2026 May 14;208(6):e00156-26. doi: 10.1128/jb.00156-26 (PMC13277301; doi:10.1128/jb.00156-26)
Supplement: Supplemental figures and tables — Fig. S1 to S7, and Tables S1 and S2. [file jb.00156-26-s0001.pdf]

Supplemental Figures and Tables for:

Adaptive evolution resulted in three subtypes of the *Riemerella anatipestifer crpR1* gene

Jialing Wang[§], Xiaoli Du[§], Hui Yin, Xiaoying Zhang, Shiqi Wang, Hongyan Liao, Xingyu Zhang, Yuting Zheng, Qinghai Hu[*]

Shanghai Veterinary Research Institute, Chinese Academy of Agricultural Sciences, 518 Ziyue Road, Shanghai 200241, China

[§]These authors contributed equally to this work.

Address correspondence to Qinghai Hu, huqinghai@caas.cn

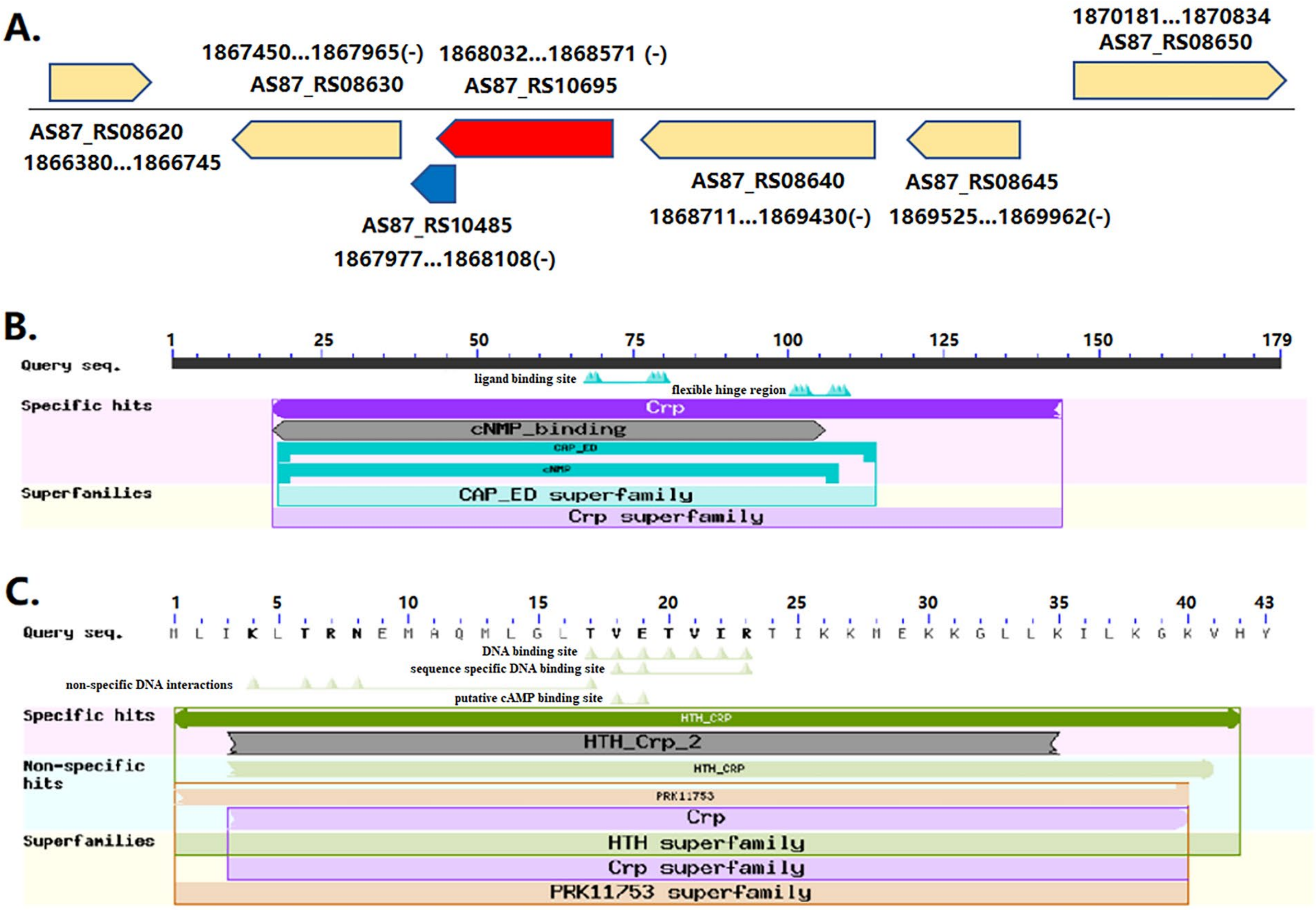

A.
AS87_RS08620
1866380...1866745
1867450...1867965(-)
AS87_RS08630
AS87_RS10485
1867977...1868108(-)
1868032...1868571 (-)
AS87_RS10695
AS87_RS08640
1868711...1869430(-)
AS87_RS08645
1869525...1869962(-)
1870181...1870834
AS87_RS08650
B.
Query seq.
ligand binding site
flexible hinge region
Specific hits
Crp
cNMP_binding
CAP_ED
cNMP
Superfamilies
CAP_ED superfamily
Crp superfamily
C.
Query seq.
M L I K L T R N E M A Q M L G L T V E T V I R T I K K M E K K G L L K I L K G K V H Y
DNA binding site
sequence specific DNA binding site
non-specific DNA interactions
putative cAMP binding site
Specific hits
HTH_CRP
HTH_Crp_2
Non-specific hits
HTH_CRP
PRK11753
Crp
Superfamilies
HTH superfamily
Crp superfamily
PRK11753 superfamily

Fig. S1. Genomic location and prediction of the conserved domains in the encoded protein of Yb2 strain *crpR1* (AS87_RS10695) and downstream AS87_RS10485. (A) The *crpR1* genes and upstream and downstream genes of the genome of strain Yb2. There was an overlap of 77 nucleotides between the 3' end of the *crpR1* ORF and the 5' end of the AS87_RS10485 ORF. (B) The CrpR1 protein of strain Yb2 only contained a CRP domain. (C) AS87_RS10485 encoded CRP-related HTH motifs.

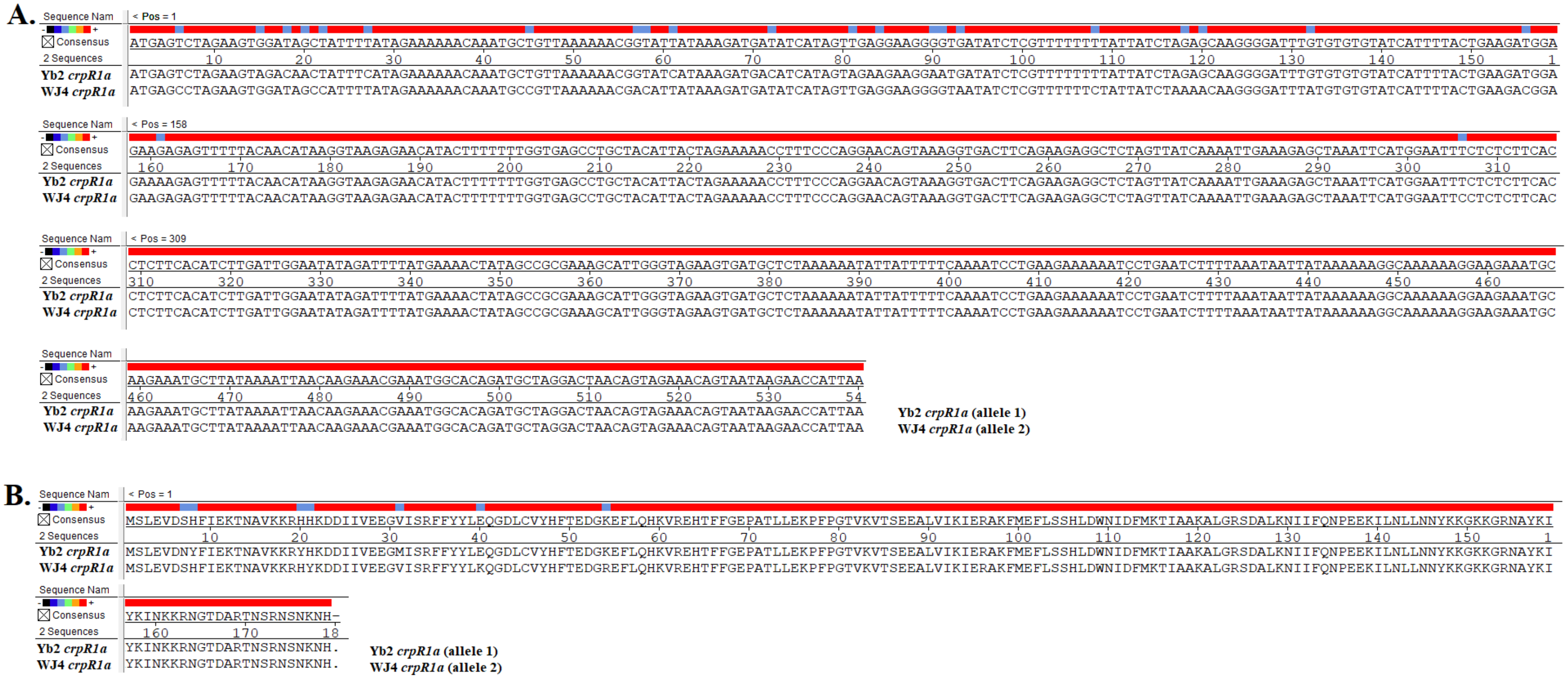

Fig. S2. Nucleotide and amino acid homology of allele 1 and allele 2 of *R. anatipestifer crpR1a.* The ClustalW method with Lasergene software 7.01 was used to assess the homology of the *crpR1a* sequences of strains Yb2 and WJ4 as representatives of allele 1 and allele 2, respectively. There were 23 base substitutions and seven amino acid differences between the two *crpR1a* alleles, primarily located at the 5' end. (A) Nucleotide homology. (B) Amino acide homology.

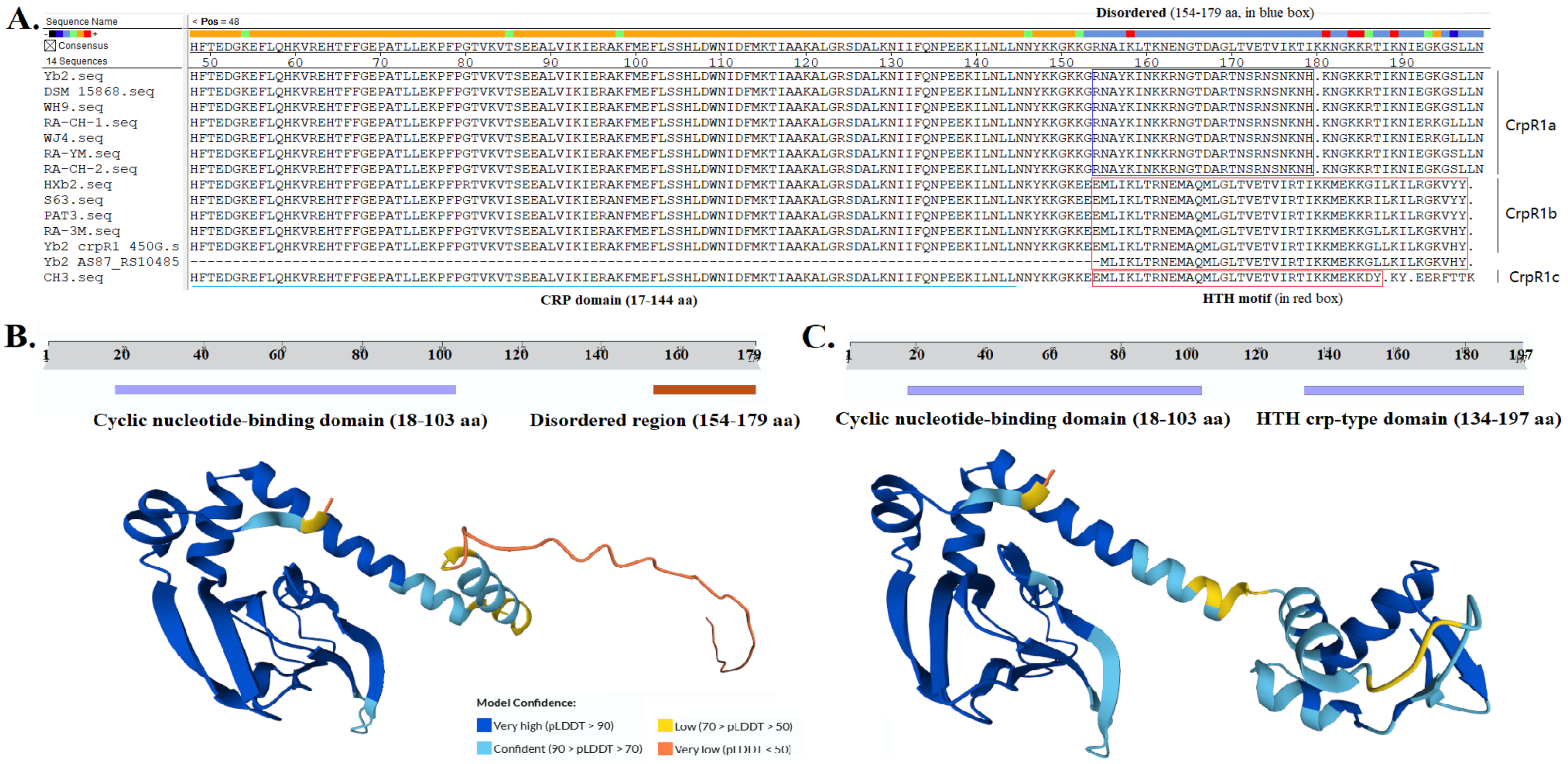

Fig. S3. Structural differences of three subtypes of *R. anatipestifer crpR1* encoded proteins. (A) The CrpR1b and CrpR1c proteins of *R. anatipestifer* strains contain both the CRP domain and HTH motif as the classical CRP protein, while the CrpR1a protein only had the CRP domain, and a disordered region at the C-terminal. (B) Predicted 3D structure of CrpR1 proteins of strain Yb2 and HXb2. The 3D structures of

CrpR1 proteins of *R. anatipestifer* strains Yb2 and HXb2 were searched against the UniprotKB with 3D structure predictions (AlphaFold) as the target database using BLAST tool with default parameters. The 3D structure of Yb2 CrpR1 was identical (100% amino acid homology) to that of the type strain ATCC 11845 (DSM 15868).

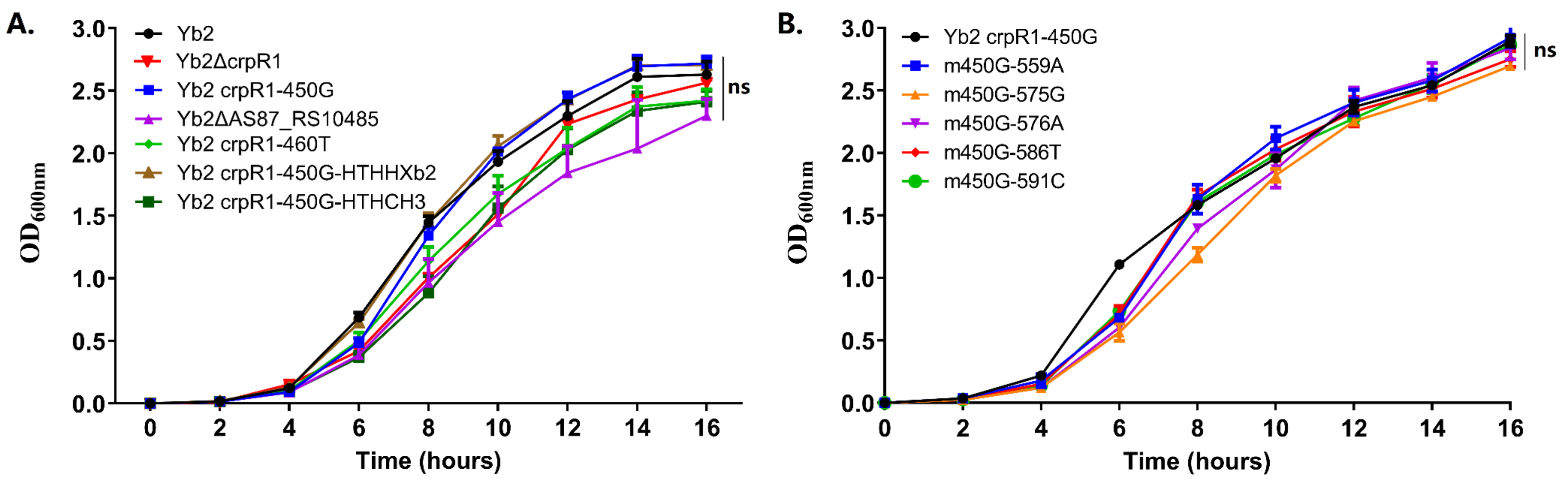

Fig. S4. Growth curves in TSB of the wild-type Yb2, the deletion mutant Yb2ΔcrpR1 and 10 mutants with point mutations. (A) The growth curves of the wild-type Yb2, the deletion mutant Yb2ΔcrpR1, and mutant strains with point mutations Yb2 crpR1-450G, Yb2ΔAS87_10485, Yb2 crpR1-460T, Yb2 crpR1-450G-HTH$_{HXb2}$, and Yb2 crpR1-450G-HTH$_{CH3}$. (B) The growth curves of the mutant Yb2 crpR1-450G and its derived five mutants with point mutations. There was no significantly difference in the growth curves among the wild-type Yb2, the deletion mutant Yb2ΔcrpR1 and 10 mutants with point mutations.

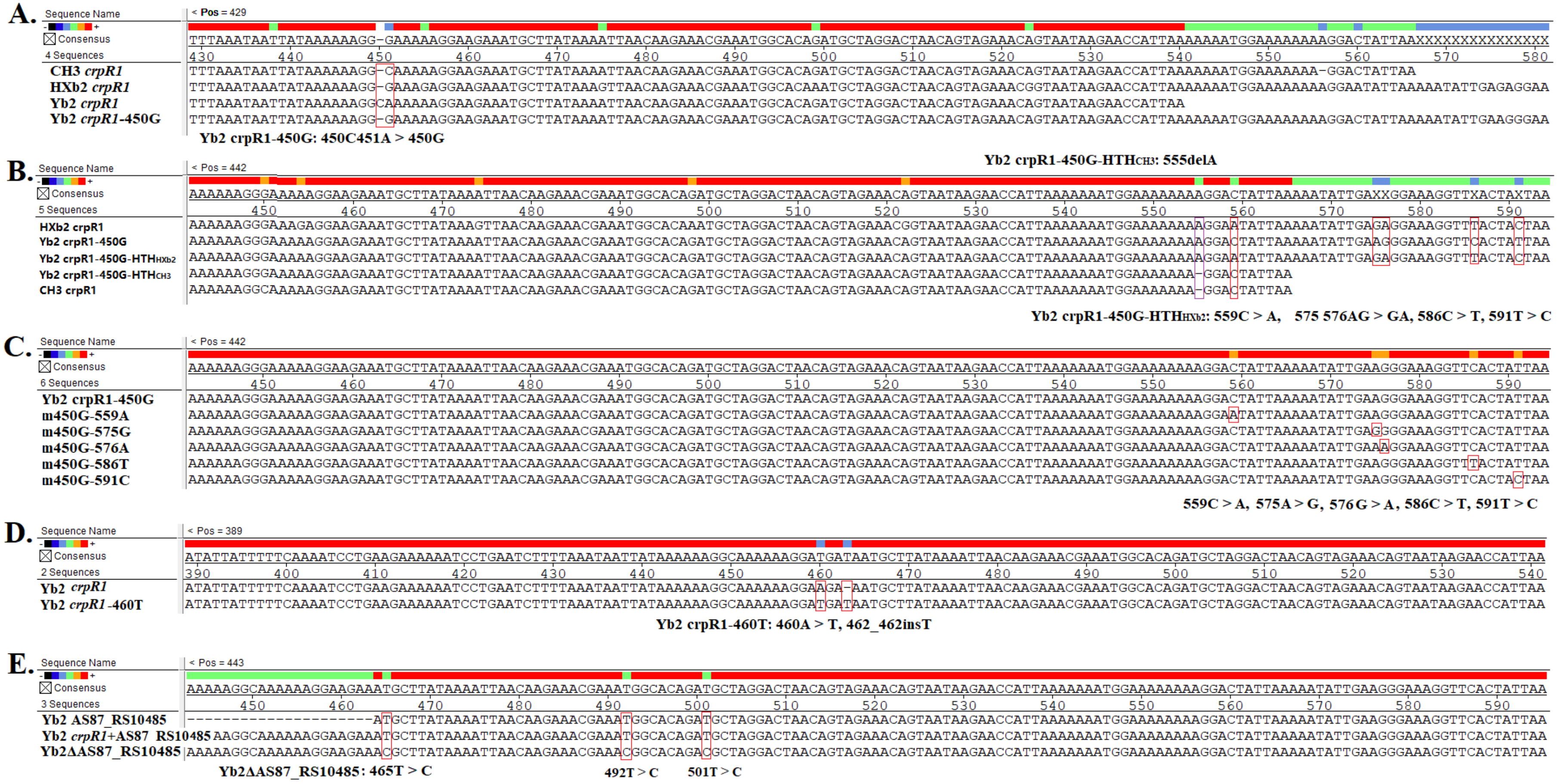

Fig. S5. Mutation sites and construction strategies of point mutant strains constructed in this study.

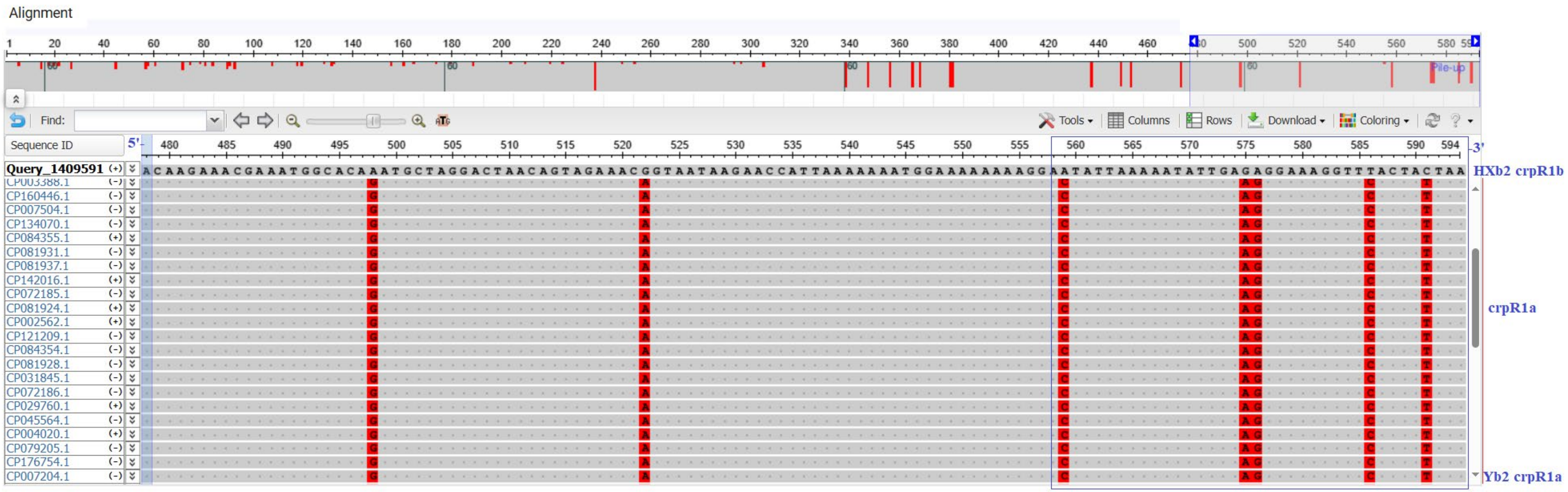

Fig. S6. Five nucleotide replacement at 3'-end of *crpR1* ORF are common between *crpR1b* (carried by HXb2) and *crpR1a* (Yb2 and other strains).

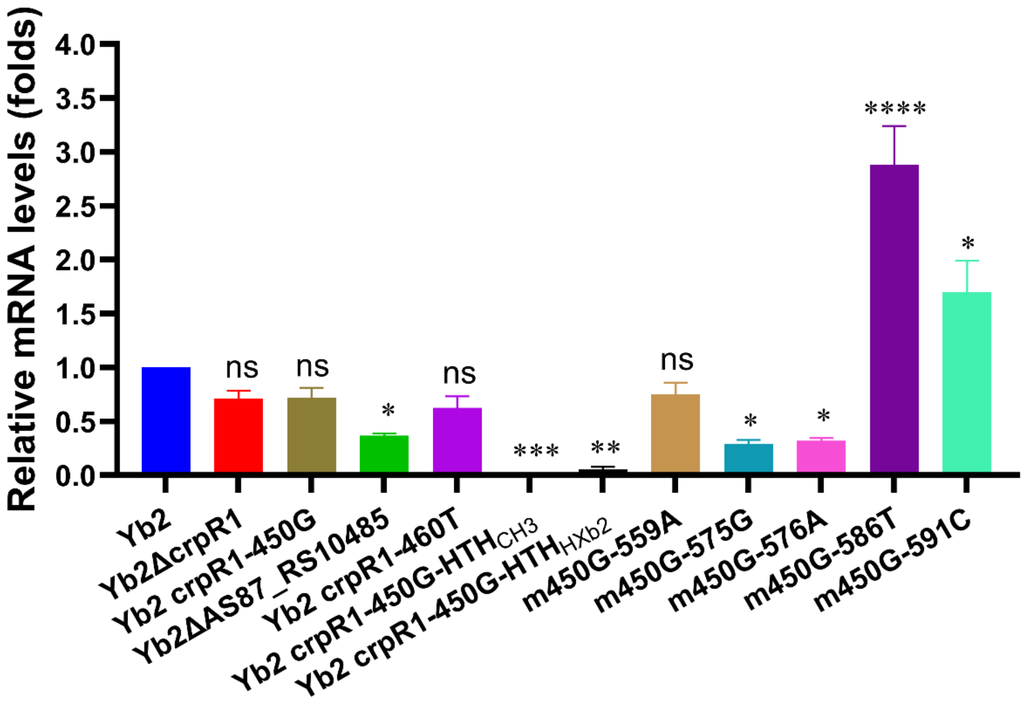

Fig. S7. Relative mRNA levels of AS87_RS10485 (encoding HTH) in the wild-type Yb2, the deletion mutant Yb2ΔcrpR1 and 10 mutants with point mutations as determined by real-time PCR. The experiment was repeated three times. Asterisks indicate statistically significant differences compared to that of the wild type Yb2 (*$p < 0.05$; **$p < 0.01$; ***$p < 0.001$; ****$p < 0.0001$; ns, no significant difference).

Table S1. *3JFNFSFMMB BOBUJQFTUJGFS* **strains whose genomes are available in GenBank** （up to January 1, 2025）

***Riemerella anatipestifer* strains**	**Accession No.[a]**	**Collection date (year)**	**Isolation site**	**Percent Identity[b]**	**Locus tag of *crpR1***	**Locus tag for downstream ORF encoding HTH motif**
Yb2	CP007204.1	2000	China: Jiangsu	100.00%	AS87_RS10695 / AS87_08965	AS87_RS10815
DSM 15868	CP002346.1	1954	USA: Long Island	100.00%	Riean_1679	
ATCC 11845 = DSM 15868=NCTC11014	CP003388.1	1954	USA: Long Island	100.00%	RA0C_1975	
NCTC11014	LT906475.1	1954	USA: Long Island	100.00%	SAMEA4063029_01740	SAMEA4063029_01739
RA-GD	CP002562.1		China: Guangdong	100.00%	RIA_0506	
WH9	CP033039.1	2016	China: Henan	100.00%	D9O39_11395	D9O39_11390
20190403E1-1	CP072186.1	2019	China: Jiangsu	100.00%	J6M13_08115	J6M13_08110
SX-1	CP134070.1	2020	China	100.00%	RI061_04640	
RCAD0125	CP121209.1	2012	China: Hainan	100.00%	AWN91_008545	
20160930R-3	CP081927.1	2016	China: Jiangsu	100.00%	K6T45_08390	K6T45_08385
RCAD0122	CP088073.1	2012	China: Guangdong	100.00%	AWB56_008860	
20190109E1-1	CP072185.1	2019	China: Jiangsu	100.00%	J6L78_08625	J6L78_08620
20190212E1-4	CP081924.1	2019	China: Jiangsu	100.00%	K6T41_08325	K6T41_08330
20190507E1-1	CP081934.1	2019	China: Jiangsu	100.00%	K6T43_02725	K6T43_02730
RA-LZ01	CP045564.1	2012	China: Guangdong	100.00%		
RA-PNBC-21-0706-PC	CP084354.1	2021	Canada: British Columbia	100.00%	LEQ05_01950	LEQ05_01945

RA-YM	CP079205.1	2001	China: Hubei	100.00%	KYF39_08535	KYF39_08530
59RAST107	CP142016.1	2023	China: Fuyang	100.00%	VIX88_02730	
RA-CH-2	CP004020.1		China	100.00%	G148_1907	
153	CP007504.1		China	100.00%	CG09_1858	
20190121E1-3	CP072188.1	2019	China: Jiangsu	100.00%	J6M00_02045	J6M00_02050
20190510E1-1	CP081928.1	2019	China: Jiangsu	100.00%	K6T46_08470	K6T46_08465
RCAD0569	CP095467.1	2017	China: Beijing	100.00%	MWN60_08500	
20190609E1-1	CP081937.1	2019	China: Jiangsu	100.00%	K6T44_08100	K6T44_08095
RCAD1242	CP168321.1	2020	China: Henan	100.00%	PG338_008830	PG338_008825
RCAD0133	CP029760.1	2011	China: Chongzhou	100.00%	AWB57_02340	AWB57_02345
RA-NM	CP160446.1	2020	China: Jiangsu	100.00%		
RA-PNBC-21-0706-AS	CP084355.1	2021	Canada: British Columbia	100.00%	LEQ03_00055	LEQ03_00060
TLb2	CP159333.1	2013	China: Shanghai	100.00%	ABU610_08550	ABU610_08545
20190509E1-1	CP081925.1	2019	China: Jiangsu	100.00%	K6T42_02895	K6T42_02900
SCVM0004	CP104076.1	2018	China: Chongqing	100.00%	N1F80_08960	
20190305E2-2	CP072190.1	2019	China: Jiangsu	100.00%	J6M09_02290	J6M09_02295
20200501E2-1	CP081931.1	2020	China: Jiangsu	100.00%	K6T47_08190	K6T47_08185
RCAD0392	CP031845.1	1998	China: Sichuan	100.00%	D1J34_02735	D1J34_02730
RCAD0416[c]	CP073239.1	2017	China: Sichuan	99.16%	D1J36_000945 (594 bp)	
RA-3M	CP144351.1	2023	China: Kunming	98.32%	V2H88_08605 (594 bp)	
20190213Y1-1	CP081929.1	2019	China: Jiangsu	98.32%	K6T48_08175 (594 bp)	
HXb2	CP011859.1	2000	China: Anhui	96.64%	AB406_1586 (594 bp)	
RA-CH-1	CP003787.1		China: Sichuan	95.46%	B739_0182	
RCAD0511	CP095466.1	2017	China: Sichuan	95.46%	MWN59_09645	
RCAD0509	CP094374.1	2017	China: Tibet	95.46%	MPN29_09650	
20190604J2-1	CP072196.1	2019	China: Jiangsu	95.46%	J6342_09355	
WJ4	CP041029.1	2000	China: Jiangsu	95.46%	FIP52_09345	

RCAD0510	CP095465.1	2017	China: Chongqing	95.46%	MWN58_09650	
XG19	CP076675.1	2019	China: Nanchang	95.46%	KPF23_02180	
20160930RA1	CP081923.1	2016	China: Jiangsu	95.46%	K6T40_09730	K6T40_09725
CH3	CP006649.1	2000	China: Jiangsu	95.13%	M949_0619 (564 bp)	
RCAD0866	CP168322.1	2018	China: Guangdong	92.61%	PGK76_008950 (594 bp)	
S63	CP110126.1	2020	China	92.61%	OIS45_07655 (594 bp)	
RCAD0421	CP121210.1	2017	China: Guangdong	92.61%	D1Y77_008610 (594 bp)	
PAT3	CP170447.1	2024	China: Fujian	92.61%	ACFKQE_09325 (594 bp)	

[a] GenBank accession number of complete genome or genome assembly.

[b] The nucleotide sequences of *crpR1* ORF were compared with that of strain Yb2.

[c] The red font indicates the CrpR1 protein in these strains has both CRP domain and HTH motif.

Table S2. The primers used in this study

Primers	Description[a]	Source or reference
**Primers for construction of the deletion mutant Yb2ΔcrpR1**		
crpR1-L-P1	5' CGGGGGATCCCTAGGTATTATAGCTATCATTGGGTGTAAT 3'	this study
crpR1-L-P2	5' GAGTTTTTAGAAAAAATCCTGAATCTTTTAAATAATTATAA 3'	this study
crpR1-R-P1	5' TCTAAAAACTCTTTTCCATCTTCAGTAAAAT 3'	this study
crpR1-R-P2	5' CCAAGCTTGCATGCTAAATAGGTAACCCTGCACTACCAGA 3'	this study
**Primers for construction of the mutant Yb2 crpR1-450G**		
Yb2-450G-L-P1	5' GCGGAAAAATTCGGGGGATCCAATAATTGCTAATTTTCAAATAAATTCATT 3'	this study
Yb2-450G-L-P2	5' GCATTTCTTCCTTTTTCCCTTTTTTATAATTATTTAAAAGATTCA 3'	this study
Yb2-450G-R-P1	5' AGGGAAAAAGGAAGAAATGCTTATAAAATTAACAA 3'	this study
Yb2-450G-R-P2	5' GATTACGCCAAGCTTGCATGCTTTGCCTGTGTAGTAGCAGGAGTATT 3'	this study
**Primers for construction of the mutant Yb2 crpR1-450G-HTH$_{HXb2}$**		
450G-HTH$_{HXb2}$-L-P1	5' GCGGAAAAATTCGGGGGATCCAATATTAAAAATATTGAGAGGAAAGGTTTACTACTA 3'	this study
450G-HTH$_{HXb2}$-L-P2	5' TTTGCCTGTGTAGTAGCAGGAGTATT 3'	this study
450G-HTH$_{HXb2}$-R-P1	5' CCTGCTACTACACAGGCAAAAAACAGGTATACTATTTTCATAAAACTATGGT 3'	this study
450G-HTH$_{HXb2}$-R-P2	5' GATTACGCCAAGCTTGCATGCTATCTACTTTTACAAAGGAAATTACGCC 3'	this study
**Primers for construction of the mutant Yb2 crpR1-450G-HTH$_{CH3}$**		

450G-HTH$_{CH3}$-L-P1	5' GCGGAAAAATTCGGGGGATCCAATAATTGCTAATTTTCAAATAAATTCATT 3'	this study
450G-HTH$_{CH3}$-L-P2	5' TTAATAGTCCTTTTTTTCCATTTTTTTAATGGTTCT 3'	this study
450G-HTH$_{CH3}$-R-P1	5' TGGAAAAAAAGGACTATTAAAAATATTGAGAGGAAAGG 3'	this study
450G-HTH$_{CH3}$-R-P2	5' GATTACGCCAAGCTTGCATGCAAAATTAACCCTGTTTTGACTATTTTAGA 3'	this study
**Primers for construction of the mutant Yb2 crpR1-460T**		
460T-463T-L-P1	5' GCGGAAAAATTCGGGGGATCCAATAATTGCTAATTTTCAAATAAATTCATT 3'	this study
460T-463T-L-P2	5' GCATTATCATCCTTTTTTGCCTTTTTTATAATT 3'	this study
460T-463T-R-P1	5' GCAAAAAAGGATGATAATGCTTATAAAATTAACAAGAAACGAA 3'	this study
460T-463T-R-P2	5' GATTACGCCAAGCTTGCATGCAAAATTAACCCTGTTTTGACTATTTTAGA 3'	this study
**Primers for construction of the mutant Yb2 crpR1-mHTH**		
mAS87_RS10485-L-P1	5' GCGGAAAAATTCGGGGGATCCGCTAATTTTCAAATAAATTCATTTAAGATAA 3'	this study
mAS87_ RS10485-L-P2	5' ATTTTATAAGCGTTTCTTCCTTTTTTGCCTTTTTTATAATTATTTAAAAGA 3'	this study
mAS87_ RS10485-R-P1	5' AAAAGGCAAAAAAGGAAGAAACGCTTATAAAATTAACAAGAAACGAAACGGCACAGACG 3'	this study
mAS87_ RS10485-R-P2	5' CTTGCATGCCTGCAGGTCGACGTCTAGCTAAAGAAAGTAATATTATGCTTGA 3'	this study
**Primers for construction of the mutant m450G-559A**		
m450G-559A-L-P1	5' GCGGAAAAATTCGGGGGATCCAAATATAATGTTCTCCAAAGCCTGTG 3'	this study
m450G-559A-L-P2	5' TTAATATTCCTTTTTTTTCCATTTTTTTAATG 3'	this study
m450G-559A-R-P1	5' GGAAAAAAAAGGAATATTAAAAATATTGAAGGGAAAGGTTCA 3'	this study
m450G-559A-R-P2	5' GATTACGCCAAGCTTGCATGCATTATGAAAAAAATATTGGTTTATACACTAGG 3'	this study
**Primers for construction of the mutant m450G-575G**		
m450G-575G-L-P1	5' GCGGAAAAATTCGGGGGATCCAAATATAATGTTCTCCAAAGCCTGTG 3'	this study

m450G-575G -L-P2	5' GTGAACCTTTCCCCTCAATATTTTAATAGTCCTTTTTTTC 3'	this study
m450G-575G -R-P1	5' TATTGAGGGGAAAGGTTCACTATTAAAAAGACTGTAC 3'	this study
m450G-575G -R-P2	5' GATTACGCCAAGCTTGCATGCATTATGAAAAAAATATTGGTTTATACACTAGG 3'	this study
**Primers for construction of the mutant m450G-576A**		
m450G-576A-L-P1	5' GCGGAAAAATTCGGGGGATCCAAATATAATGTTCTCCAAAGCCTGTG 3'	this study
m450G-576A -L-P2	5' GTGAACCTTTCCTTTCAATATTTTAATAGTCCTTTTTTTC 3'	this study
m450G-576A -R-P1	5' TATTGAAAGGAAAGGTTCACTATTAAAAAGACTGTACAT 3'	this study
m450G-576A -R-P2	5' GATTACGCCAAGCTTGCATGCATTATGAAAAAAATATTGGTTTATACACTAGG 3'	this study
**Primers for construction of the mutant m450G-586T**		
m450G-586T-L-P1	5' GCGGAAAAATTCGGGGGATCCAAATATAATGTTCTCCAAAGCCTGTG 3'	this study
m450G-586T -L-P2	5' AATAGTAAACCTTTCCCTTCAATATTTTAATAG 3'	this study
m450G-586T -R-P1	5' GAAGGGAAAGGTTTACTATTAAAAAGACTGTACATGAATAAAATATCA 3'	this study
m450G-586T -R-P2	5' GATTACGCCAAGCTTGCATGCATTATGAAAAAAATATTGGTTTATACACTAGG 3'	this study
**Primers for construction of the mutant m450G-591C**		
m450G-591C-L-P1	5' GCGGAAAAATTCGGGGGATCCAAATATAATGTTCTCCAAAGCCTGTG 3'	this study
m450G-591C -L-P2	5' AGTAGTGAACCTTTCCCTTCAATATTT 3'	this study
m450G-591C -R-P1	5' GAAGGGAAAGGTTCACTACTAAAAAGACTGTACATGAATAAAATATCAGATA 3'	this study
m450G-591C -R-P2	5' GATTACGCCAAGCTTGCATGCATTATGAAAAAAATATTGGTTTATACACTAGG 3'	this study
**Primers for identification of the deletion mutant Yb2ΔcrpR1**		
crpR1-id-P1	5' AGCAACTCCACTAGTATGAGTCTAGAAGTAGACAACTATTTCATA 3'	this study
crpR1-id-P2	5' TCCGCATGCTTAATGGTTCTTATTACTGTTTCTACTGTTAG 3'	this study

16S rRNA P1	5' CAGCTTAACTGTAGAACTGC 3'	(47)
16S rRNA P2	5' TCGAGATTTGCATCACTTCG 3'	(47)
ErmF P1	5' GCCCGAAATGTTCAAGTTGT 3'	(47)
ErmF P2	5' TTTCCGAAATTGACCTGACC 3'	(47)
**Sequencing primers for identification of the mutants with point mutation(s)**		
mcrpR1-id P1	5' GAGCAAGGGGATTTGTGTGT 3'	this study
mcrpR1 -id P2	5' CCCAGCCATAGAAAGCAAAA 3'	this study
**Real-time PCR primers for detecting *crpR1* and its neighbor genes**		
crpR1-RT P1	5' GGGATTTGTGTGTGTATCATTTTACTG 3'	this study
crpR1-RT P2	5' TCTAGTAATGTAGCAGGCTCACCAA 3'	this study
AS87_RS10485-RT P1	5' ACGAAATGGCACAGATGCTA 3'	this study
AS87_RS10485-RT P2	5' TGAACCTTTCCCTTCAATATTTTT 3'	this study
AS87_08960-RT P1	5' TTTTGCTTTCTATGGCTGGGTAA 3'	this study
AS87_08960-RT P2	5' GGTTTTGTATGTATGCAACTGTTTGG 3'	this study
AS87_08970-RT P1	5' TAAGAGCCATGGTAAACACCAAAA 3'	this study
AS87_08970-RT P2	5' TCGTGTTCGTGCTTCATCATATT 3'	this study
AS87_07310-RT P1	5' AGCTAAATGGTCTCAGCTTGGAA 3' (for normalization)	this study
AS87_07310-RT P2	5' TGCAGGGCGAGTAATGGTTAA 3' (for normalization)	this study
